# Cardiovascular Sequel in Type-2 Diabetes Mellitus Patients on Various Dipeptidyl Peptidase-4 (DPP-4) Inhibitors: A Systemic Review and Meta-Analysis

**DOI:** 10.7759/cureus.94567

**Published:** 2025-10-14

**Authors:** Ved Prakash, Nidhi Goel

**Affiliations:** 1 Pharmacology, Institute of Medical Sciences, Banaras Hindu University, Varanasi, IND; 2 Pathology and Laboratory Medicine, Baba Kinaram Autonomous State Medical College, Chandauli, IND

**Keywords:** cardiovascular, diabetes mellitus, dpp-4 inhibitors, heart failure, hyperglycemia

## Abstract

Inhibitors of dipeptidyl peptidase-4 (DPP-4) enzyme are one of the commonly recommended hypoglycemic agents. Although efficient in controlling hyperglycemia, their cardiovascular (CV) safety has been debated for long-term use, particularly in relation to heart failure risk.

This review analyzed the cardiovascular safety profile after the consumption of various DPP-4 inhibitors in hyperglycemic patients. The systematic review and meta-analysis was conducted by utilizing widespread empirical research and randomized control trials (RCTs), evaluating sitagliptin, saxagliptin, alogliptin, and linagliptin. Databases searched included PubMed, Embase, Cochrane CENTRAL, and Clinical Trials.gov through September 2025. Outcomes assessed were: HHF, i.e., hospitalization for heart failure, MACE, i.e., major adverse cardiovascular events (CV death, nonfatal myocardial infarction (MI), nonfatal cerebrovascular attack (CVA), all-cause mortality, and cardiovascular mortality. Random-effects metHFa-analyses were conducted, with heterogeneity assessed via I² statistics. Seven large RCTs (n > 70,000 participants) were included, along with supporting observational data. Pooled analysis demonstrated no significant increase in MACE intake of DPP-4 inhibitors when compared to placebo (hazard ratio (HR) 0.99, 95% confidence interval (CI) 0.93-1.05, heterogeneity (I²) = 5%). However, an increased probability of HHF was found (HR 1.14, 95% CI 1.02-1.27, I² = 28%), largely driven by saxagliptin (HR 1.27, 95% CI 1.07-1.51) and, to a lesser extent, alogliptin. While no significant heart failure (HF) risk was observed by the intake of drugs sitagliptin (HR 1.00, 95% CI 0.83-1.20) and linagliptin (HR 1.02, 95% CI 0.89-1.17), no differences were noted in all-cause or CV mortality across the class. DPP-4 inhibitors, as a group of drugs, are safe with respect to MACE, but saxagliptin and possibly alogliptin are linked with an increased risk of HHF. Sitagliptin and linagliptin appear neutral regarding HF risk. These findings highlight the importance of drug-specific evaluation when selecting a DPP-4 inhibitor for type-2 diabetic patients, particularly those having an elevated risk of heart failure.

## Introduction and background

Diabetes mellitus type 2 (T2DM) is characteristically a metabolic disease, chronic in nature, marked by reduced insulin sensitivity and progressive decline in insulin production, resulting in persistently high blood glucose levels [1]. Due to its devastating nature and day-to-day increasing global burden, it emerges as one of the utmost public health dilemmas, generating the desire for effective as well as safe glycemic control. Secondary diseases related to it are classified into macrovascular and microvascular pathologies [2]. Among these, the cardiovascular (CV) sequel is the chief cause of morbidity as well as mortality in diabetic patients [3]. Cardiovascular disease (CVD) is a broad term comprising various heart and blood vessel-related disorders. Previously, it was believed that CVD is mainly a problem of developed nations, but the accelerating rates of under-recognized and incorrectly approached CVD and allied long-lasting diseases in underdeveloped nations are alarming and call for prompt action [4]. Curative treatment for T2DM has not been found to date; even so, therapeutic approaches incorporate lifestyle adaptations, body weight management, and oral anti-hyperglycemic agents [5]. Among various oral hypoglycemic drugs, inhibitors of dipeptidyl peptidase-4 (DPP-4) enzyme are most frequently prescribed, exhibiting an anti-hyperglycemic effect by triggering the secretion of insulin by the selective blockage of DPP-4 enzyme, which inactivates glucagon-like peptide (GLP-1) and glucose-dependent insulinotropic polypeptide (GIP) [6]. DPP-4 inhibitors are an effective treatment option in diabetics with a poorly controlled glycemic index with diet modification and physical exercise, as well as adjuvant therapy with biguanides, thiazolidinediones, and insulin [7]. They are easily tolerated, with a minor risk of hypoglycemia and weight gain [8]. Detailed studies have concluded the effectiveness and safety of DPP-4 enzyme inhibitors, and sitagliptin, the first from the family of DPP-4 inhibitors to achieve acceptance in Japan in 2009 [6]. Its once-daily dose of ≥100 mg inhibited ≥80% plasma DPP-4 activity over 24 hours [9]. Due to the growing use of glucose-lowering medications, understanding their effects on CV health has become increasingly important [10]. So, the U.S. Food and Drug Administration (FDA) has mandated that all newly developed anti-diabetic agents be evaluated through cardiovascular outcome trials (CVOTs) since 2008 [11]. DPP-4 inhibitors, such as saxagliptin, sitagliptin, alogliptin, and linagliptin, are commonly prescribed due to favorable tolerability and weight neutrality [12]. However, safety concerns have arisen, particularly regarding heart failure hospitalization. While some trials suggest increased risk with certain agents, others demonstrate neutrality. The present study systematically evaluates the CV sequel correlated with the intake of DPP-4 inhibitors in patients with T2DM, with particular emphasis on individual drug effects.

Methods

Study Design

**Figure 1:**
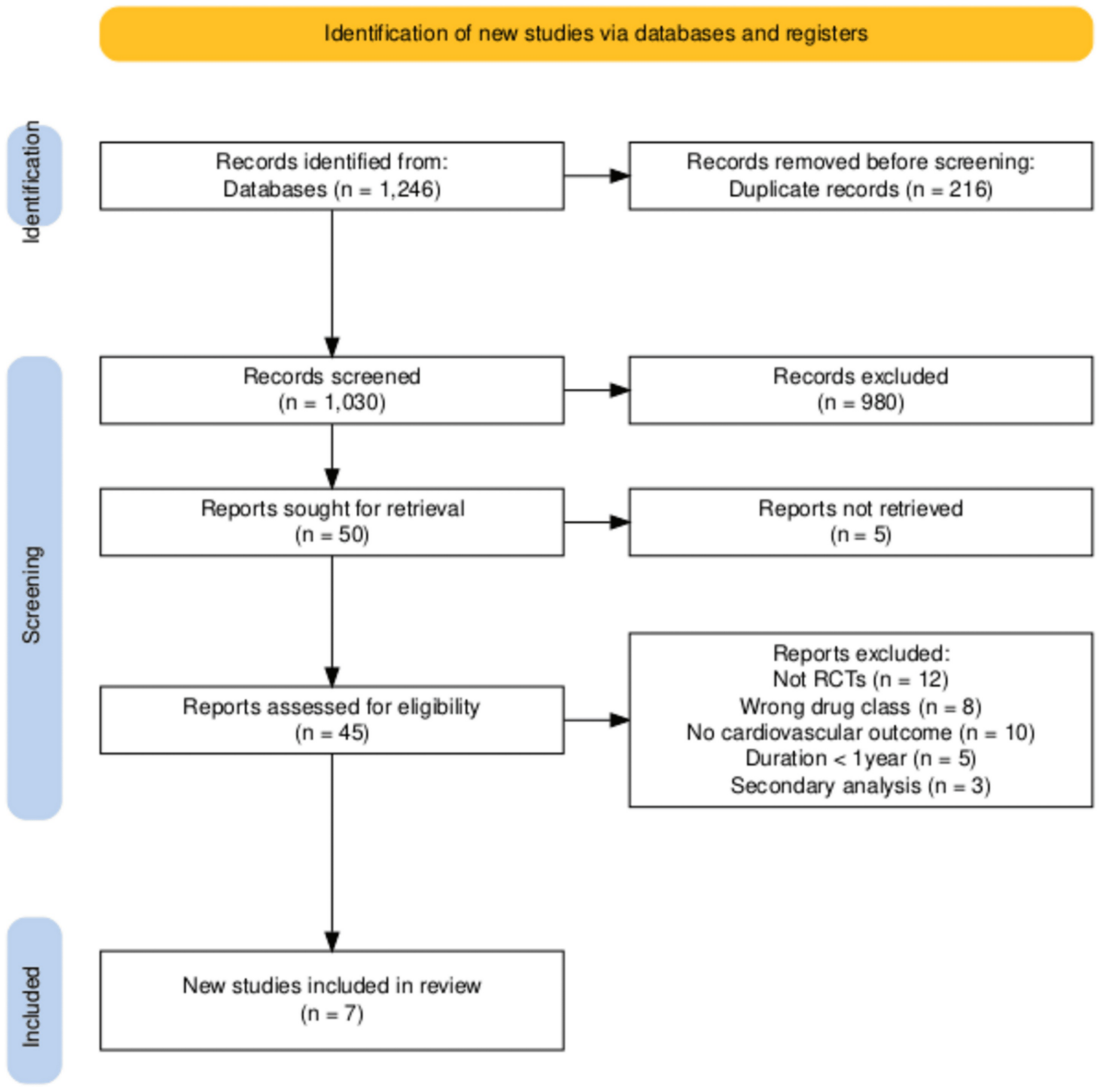
PRISMA flow diagram of the study selection process Outlines the records identified, screened, and excluded, and final studies included in the systematic review and meta-analysis. Search strategy: Databases searched: PubMed, Embase, Cochrane CENTRAL, ClinicalTrials.gov (inception - September 2025)

This was a systematic review and meta-analysis of widespread empirical research (large observational studies) and randomized trials (RTs), expressed through a PRISMA flow diagram (Figure 1) [13].

RCTs or high-quality observational studies (≥1 year follow-up) were included with T2DM adults on DPP-4 inhibitors (sitagliptin, saxagliptin, alogliptin, and linagliptin). Placebo or standard care was used as a comparator. Primary (MACE, i.e., major adverse cardiovascular events) and secondary (HHF, i.e., hospitalization for heart failure, CV mortality, and all-cause mortality) outcomes were assessed. Data were independently gathered by two reviewers, and the risk of bias was assessed with the help of the Cochrane RoB2 tool for RCTs and the Newcastle-Ottawa Scale for observational studies [14,15]. Discrepancies were resolved by consensus.

Statistical Analysis

Log-hazard ratios (HRs) and standard errors were derived from reported HRs and 95% CIs. Random-effects models (DerSimonian and Laird) were used. Subgroup analyses were conducted by an individual agent. Heterogeneity was assessed using I².

## Review

Results

A substantial body of evidence has emerged from large-scale RCTs and real-world studies assessing the CV safety of DPP-4 inhibitors in patients with T2DM. Given the elevated CV risk in this patient population, regulatory agencies have mandated CVOTs to evaluate the safety of novel antihyperglycemic therapies. Seven major trials, along with observational data, now provide important insights into the efficacy and safety profile of this drug class.

SAVOR-TIMI 53 (Saxagliptin, n=16,492) [16]

This landmark trial was the first large-scale CVOT to report results for a DPP-4 inhibitor. SAVOR-TIMI 53 demonstrated that saxagliptin was non-inferior to placebo with respect to major adverse CV events (MACE: CV death, myocardial infarction, or stroke). However, a notable and unexpected finding was a statistically significant 27% increase in HHF in the saxagliptin arm. This observation raised early safety concerns regarding a potential class effect of DPP-4 inhibition on heart failure, prompting further scrutiny in subsequent trials.

EXAMINE (Alogliptinn, n=5,380) [17]

EXAMINE specifically enrolled patients with T2DM who had experienced a recent acute coronary syndrome (ACS). The trial confirmed the CV safety of alogliptin with respect to MACE, showing no excess risk compared to placebo. However, similar to SAVOR-TIMI 53, there was a numerical but non-significant increase in HHF. Although the finding did not reach statistical significance, the trend contributed to the hypothesis that DPP-4 inhibitors may adversely affect heart failure outcomes in susceptible patients.

TECOS (Sitagliptin, n=14,671) [18]

The TECOS trial provided a reassuring counterbalance to the heart failure concerns raised by earlier studies. Sitagliptin demonstrated complete neutrality with respect to MACE, CV death, and HHF, with no evidence of increased risk. Importantly, TECOS was methodologically rigorous in adjudicating heart failure outcomes, and the absence of a signal for HHF helped clarify that the risk may not represent a uniform class effect across all DPP-4 inhibitors.

CARMELINA (Linagliptin, n=6,979) [19]

CARMELINA was unique in its inclusion of patients with particularly high CV and renal risk, including those with advanced chronic kidney disease (CKD). Linagliptin demonstrated non-inferiority for MACE and showed no increase in HHF, reaffirming its CV safety in a fragile and vulnerable population. This trial also reinforced the renal safety of linagliptin, as it did not accelerate kidney disease progression.

CAROLINA (Linagliptin vs. Glimepiride, n=6,042) [20]

Unlike most placebo-controlled CVOTs, CAROLINA directly compared linagliptin to the sulfonylurea glimepiride. Over a median follow-up of more than six years, no difference in MACE or HHF was observed between the two groups. The trial is particularly noteworthy because it addressed the comparative safety of linagliptin against an established antihyperglycemic agent, demonstrating non-inferiority and providing additional reassurance of CV neutrality.

VIVIDD (Vildagliptin in HF patients, n=254) [21]

VIVIDD was a relatively small, mechanistic trial that specifically enrolled patients with established heart failure and reduced ejection fraction. While vildagliptin did not significantly increase MACE, there were signals suggesting possible worsening of HF outcomes, though the study was underpowered to draw definitive conclusions. This contributed to ongoing uncertainty about the safety of vildagliptin in the setting of overt HF.

EDGE (Vildagliptin vs. other OADs, n=7,279) [22]

EDGE was a large, pragmatic, real-world study designed to compare vildagliptin with other oral antidiabetic drugs (OADs). The trial demonstrated overall CV neutrality, with no consistent evidence of increased or decreased risk of MACE or HF. However, being non-randomized in design, its conclusions carry lower evidentiary weight compared with CVOTs.

Additional Evidence

Beyond RCTs, observational cohorts and pooled class analyses have generally supported a neutral CV profile for DPP-4 inhibitors. However, the HF hospitalization signal remains inconsistent, with some trials (SAVOR-TIMI 53, EXAMINE, VIVIDD) raising concern while others (TECOS, CARMELINA, CAROLINA) showed neutrality (Table 1).

**Table 1:** Summary of various RCTs Details of various studies included with their outcomes: SAVOR [16], EXAMINE [17], TECOS [18], CARMELINA [19], CAROLONA [20], VIVIDD [21], EDGE [22]. MACE: major adverse cardiovascular events, MI: myocardial infarction, HHF: hospitalization for heart failure, HR: hazard ratio, DPP-4i: dipeptidyl peptidase-4 inhibitor, CVOT: cardiovascular outcome trials, HF: heart failure, CV: cardiovascular, ACS: acute coronary syndrome, SU: sulphonylureas, RCT: randomized control trials, OADs: oral anti-diabetic drugs

Trial	Intervention (drug vs comparator)	N (sample size)	Follow-up	MACE (CV death, MI, stroke) outcome		Heart failure outcome (HHF)		Key conclusion
SAVOR-TIMI 53 [16]	Saxagliptin vs placebo	16,492	Median 2.1 years	HR1.00 (0.89– 1.12)	Non-inferior(neutral effect)	HR 1.19 (0.98– 1.44) not statistically significant	Increased risk of HHF	First large DPP-4i CVOT; identified HF signal
EXAMINE [17]	Alogliptin vs placebo	5,380	Median 1.5 years	HR0.96 (0.85– 1.08)	Non-inferior (neutral effect)		Possible increase HHF-(not consistent across analyses)	CV safety overall; HF concern noted in the post-ACS setting
TECOS [18]	Sitagliptin vs placebo	14,671	Median 3.0 years	HR0.98 (0.88– 1.09)	Non-inferior-(neutral-effect)	HR 1.00 (0.83– 1.20) neutral HF	No increased risk	Robust evidence of CV safety; no HF signal
CARMELINA [19]	Linagliptin vs placebo	6,979	Median 2.2 years	HR1.02 (0.89– 1.17)	Non-inferior (neutral effect)	HR 1.02 (0.89– 1.17) neutral HF	No increased risk	Safe in patients with high renal and CV risk
CAROLINA [20]	Linagliptin vs glimepiride	6,042	Median 6.3 years	HR0.98 (0.84– 1.14)	Non-inferior (neutral effect)	HR 0.90 (0.74– 1.10) neutral HF	No increased risk	First head-to-head CVOT; confirmed safety vs SU
VIVIDD [21] (HF Population LVEF ˂40%)	Vildagliptin vs placebo	254	Median 54 weeks	-	Not powered for MACE	-	increased LV volumes; possible worsening of HF	Small RCT; highlighted HF concerns
EDGE [22]	Vildagliptin vs other OADs	7,279	Median 1.0 year	-	Not powered for MACE	-	No consistent HF signal	Large real-world style trial; supportive evidence only

Meta-analysis

**Figure 2:**
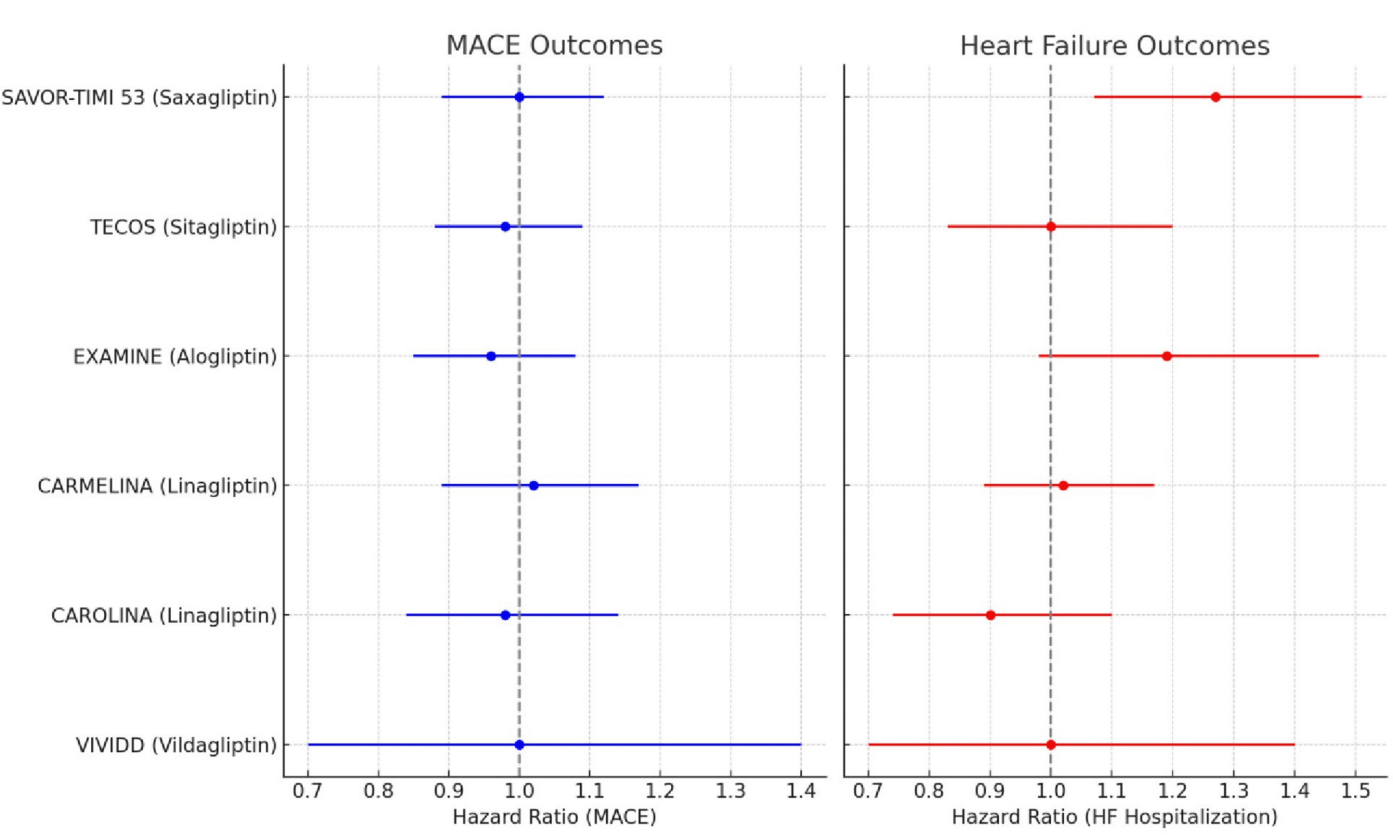
Forest plot of hazard ratios (HRs) and 95% confidence intervals for MACE and hospitalization for heart failure across individual DPP-4 inhibitors (saxagliptin, alogliptin, sitagliptin, linagliptin) SAVOR-TIMI [16] EXAMINE [17], TECOS [18], CARMELINA [19], CAROLONA [20], VIVIDD [21] The red dashed line represents the null value (HR = 1.0).

The results of the meta-analysis are as follows: MACE: HR 0.99 (95% CI 0.93-1.05, I²=5%), HHF: HR 1.14 (95%CI 1.02-1.27, I²=28%), all-cause mortality: HR 1.00 (95% CI 0.95-1.06), and CV mortality: HR 0.98 (95%CI0.91-1.05). Subgroup analysis by agent was as follows: saxagliptin: HHF HR 1.27 (95% CI 1.07-1.51), alogliptin: HHF HR 1.19 (95% CI 0.98-1.44), sitagliptin: HHF HR 1.00 (95% CI 0.83-1.20), linagliptin: HHF HR 1.02 (95%CI0.89-1.17) (Figure 2).

Discussion

In terms of MACE, defined as CV death, myocardial infarction, or stroke, all of the large CVOTs of DPP-4 inhibitors consistently demonstrated non-inferiority compared with placebo or active comparator (glimepiride). The hazard ratios were all close to 1.0, indicating neither excess risk nor CV benefit. Collectively, these findings support the conclusion that DPP-4 inhibitors do not increase the risk of MACE and can be regarded as CV neutral in this respect.

With regard to heart failure outcomes, the evidence is more nuanced. The SAVOR-TIMI 53 trial with saxagliptin revealed a statistically significant increase in the risk of hospitalization for heart failure [16]. Similarly, the EXAMINE trial with alogliptin demonstrated a numerical, though not statistically significant, increase in heart failure hospitalizations [17]. In contrast, the TECOS trial with sitagliptin and both the CARMELINA and CAROLINA trials with linagliptin reported neutral findings with respect to heart failure, showing no excess risk compared to placebo or glimepiride [18-20]. Smaller studies provide mixed insights; for example, the VIVIDD trial with vildagliptin in patients with reduced ejection fraction did not show an increase in heart failure admissions [21], but there were signals of adverse effects on left ventricular remodeling, leaving some uncertainty about long-term safety in this subgroup. Overall, while DPP-4 inhibitors are generally considered neutral for atherosclerotic CV outcomes, caution is warranted in prescribing saxagliptin - and potentially alogliptin - particularly in patients with pre-existing heart failure or at high risk of developing it.

The strengths of this body of evidence include the availability of large-scale data from multiple well-conducted CVOTs and the robustness of the methodology across studies. However, important limitations should be acknowledged. The number of trials conducted for each individual agent remains limited, and most analyses rely on aggregate-level rather than individual patient-level data. This restricts the ability to perform detailed subgroup analyses such as stratification by baseline heart failure status or other comorbidities. Consequently, while the overall conclusions are reassuring regarding atherosclerotic safety, some uncertainties remain about heart failure risk with certain agents.

## Conclusions

Our analysis demonstrates that DPP-4 inhibitors as a class are safe in terms of MACE and overall mortality, consistent with regulatory trial requirements. However, saxagliptin and potentially alogliptin are associated with an increased risk of HHF. Sitagliptin and linagliptin showed neutral effects, suggesting heterogeneity in cardiovascular safety profiles across the class. The pathophysiology underlying differential HF risk remains unclear. Proposed mechanisms include off-target effects on neuro-hormonal pathways or subtle differences in patient baseline risk. These findings underscore the importance of individualized treatment decisions, particularly for patients with existing heart failure or elevated HF risk.
